# Predicting isolated impaired glucose tolerance without oral glucose tolerance test using machine learning in Chinese Han men

**DOI:** 10.3389/fendo.2025.1514397

**Published:** 2025-04-24

**Authors:** Lin Wang, Jing Xie, Zhaoyan Gu, Xinyu Miao, Lichao Ma, Shuangtong Yan, Yanping Gong, Chunlin Li, Banruo Sun, Yue Ruan

**Affiliations:** ^1^ Department of Endocrinology, Second Medical Center, Chinese People’s Liberation Army General Hospital, National Clinical Research Center for Geriatric Diseases, Beijing, China; ^2^ Department of Special Medical Service, Ninth Medical Center, Chinese People’s Liberation Army General Hospital, Beijing, China; ^3^ Institute of Biomedical and Health Engineering, Shenzhen Institutes of Advanced Technology, Chinese Academy of Sciences, Shenzhen, China

**Keywords:** pre-diabetes, isolated impaired glucose tolerance, machine learning models, oral glucose tolerance test, fasting plasma glucose

## Abstract

**Background:**

Isolated Impaired Glucose Tolerance (I-IGT) represents a specific prediabetic state that typically requires a standardized oral glucose tolerance test (OGTT) for diagnosis. This study aims to predict glucose tolerance status in Chinese Han men at fasting state using machine learning (ML) models with demographic, anthropometric, and laboratory data.

**Methods:**

The study population consisted of 1,117 Chinese Han men aged 50–87 years. Baseline variables including age, fasting plasma glucose (FPG), high blood pressure (HBP), body mass index (BMI), waist to hip ratio (WHR), total cholesterol (TC), triglyceride (TG), high-density lipoprotein cholesterol (HDL-C), and low-density lipoprotein cholesterol (LDL-C) were collected from electronic medical records (EMRs) for machine learning model training and validation. Support Vector Machine (SVM), Decision Tree (DT), Random Forest (RF), Logistic Regression (LR), K-Nearest Neighbors (KNN), Naive Bayes (NB), Adaptive Boosting (AdaBoost) and Gradient Boosting Machines (GBM) were tested for machine learning model performance comparison. Model performance was evaluated using metrics including accuracy, recall, F1 score, positive predictive value (PPV), negative predictive value (NPV), and the area under the receiver operating characteristic curve (AUC). Shapley Additive Explanations (SHAP) and confusion matrix plots were used for model interpretation.

**Results:**

The RF model demonstrated the best overall performance with a 96.7% accuracy, recall of 91.4%, F1 score of 95.7%, PPV of 99.1%, and NPV of 95.6%. The AUC values for the SVM, DT, RF, LR, KNN, NB, AdaBoost, and GBM models were 0.97, 0.92, 0.96, 0.97, 0.88, 0.88, 0.97, and 0.97, respectively. While the RF model showed strong overall performance, the LR model had the highest AUC, indicating superior discriminatory power. FPG was identified as the most important predictor for I-IGT, followed by HDL, TC, HBP, BMI, and WHR. Individuals with FPG levels higher than 5.1 mmol/L were more likely to have I-IGT; the performance metrics for this cut-off value were: 89.35% accuracy, 89.79% recall, 85.22% F1 score, 81.09% PPV, 94.38% NPV, and 0.95 AUC.

**Conclusion:**

Machine learning models based on demographic and clinical characteristics offer a cost-effective method for predicting I-IGT in Chinese Han men aged over 50, without the need for an OGTT. These models could complement existing early diagnostic strategies, thereby enhancing the early detection and prevention of diabetes. Additionally, FPG alone could serve as an efficient screening tool for the early identification of I-IGT in clinical settings.

## 1 Introduction

Diabetes mellitus is a common chronic disease that afflicts millions worldwide. According to the International Diabetes Federation (IDF) Diabetes Atlas reports, 537 million adults were living with diabetes in 2021 (1). The rising prevalence of diabetes imposes a significant burden on healthcare systems and contributes to numerous complications that adversely affect human health (2). Type 2 diabetes (T2DM) constitutes over 90% of all diabetes cases globally (3). The disease spectrum of T2DM encompasses various stages, from prediabetes to T2DM with end stage complications. Prediabetes, defined by either impaired glucose tolerance (IGT) or impaired fasting glucose (IFG), significantly increase the risk of progressing to T2DM (4). IGT is characterized by elevated blood glucose levels two hours after an Oral Glucose Tolerance Test (OGTT), with or without the presence of IFG. A review study on the incidence of prediabetes among different ethnicities revealed that the prevalence of IFG was 48.1% in Asians, while IGT was observed in 27.7% of the Asian population (5). Recent epidemiological data indicate that the prevalence of prediabetes among Chinese men and women are 37.0% and 33.4%, respectively. Men have higher prevalence than women for both prediabetes and diabetes. Among the five ethnic groups surveyed in China, the Han ethnic group exhibits the highest prevalence rate of diabetes (6). Isolated Impaired Glucose Tolerance (I-IGT) specifically refers to individuals who exhibit IGT without concurrent IFG. Similar to IGT, individuals with I-IGT are also at increased risk of developing T2DM, heart disease, and stroke (7, 8). The transition from I-IGT to T2DM is particularly concerning due to the significant rise in morbidity and mortality from diabetes-related complications. Persistent hyperglycemia can also lead to chronic damage and dysfunction of various organs, including the eyes, kidneys, nerves, heart, and blood vessels (9). Additionally, I-IGT is often accompanied by other cardiovascular risk factors such as dyslipidemia, hypertension, and obesity, which collectively exacerbate the risk of cardiovascular diseases (10). The identification of I-IGT relies on OGTT, a method both time-consuming and less adaptable to large-scale screening. Additionally, many individuals with I-IGT are asymptomatic, making it less likely for them to undergo testing until more severe symptoms or complications arise. The lack of specific symptoms and the transient nature of glucose levels, which may return to normal ranges, further complicate the identification of I-IGT in the general population. HbA1c is also used to diagnose patients with prediabetes, but this standard cannot solely be used to identify individuals with IGT or I-IGT. A meta-analysis revealed that HbA1C is not a reliable marker for prediabetes detection, demonstrating a mean sensitivity of 49% and a specificity of 79% in identifying prediabetes (11). Efforts to improve the prediction of individuals with I-IGT are crucial for early intervention and prevention of diabetes and its associated complications. Recent studies have demonstrated the efficacy of machine learning (ML) models in predicting T2DM by utilizing a range of clinical, biochemical, and demographic data, such as support vector machine (SVM), decision tree (DT), logistic regression (LR)and so on (12). Duygu and Esin developed a system named LDA-MWSVM for predicting diabetes. This system uses Linear Discriminant Analysis (LDA) for reducing dimensions and extracting important features (13). To handle datasets with many dimensions, Razavian and colleagues created prediction models using logistic regression to forecast various stages of T2DM development (14). However, few studies focus on using machine learning models to identify individuals in the prediabetic stage, especially those with I-IGT.

In this study, we investigate the capability of various machine learning algorithms to predict individuals with I-IGT among those with normal fasting plasma glucose (FPG). Our objective is to equip healthcare professionals with a more targeted and cost-effective approach to I-IGT detection and management, thereby enhancing patient care quality and health outcomes.

## 2 Materials and methods

### 2.1 Study subjects

We conducted a retrospective study using data from male individuals who underwent medical examinations at the Chinese People’s Liberation Army General Hospital from May 1998 to August 2005. Participants were included if they were Chinese Han men with normal FPG. Exclusion criteria encompassed individuals with abnormal FPG, diagnosed diabetes, conditions affecting glucose tolerance, or those on medications influencing the results of OGTT. Ultimately, 1,117 participants met the criteria.

### 2.2 Data collection

All participants underwent comprehensive blood tests and physical examinations in an outpatient setting. Data on height, weight, waist and hip circumferences, blood pressure, and other pertinent parameters were documented.

#### 2.2.1 Biochemical analyses

Serum lipid profiles, including triglyceride (TG), total cholesterol (TC), high-density lipoprotein cholesterol (HDL-C), and low-density lipoprotein cholesterol (LDL-C) levels, were determined using chemiluminescence on an automated analyzer. All participants underwent an OGTT, with venous plasma glucose measurements taken before (FPG) and 2 hours post-OGTT. The enzymatic hexokinase method was utilized for FPG and 2-hour post-glucose (2 h-PG) level determinations.

#### 2.2.2 Glucose tolerance categorization

Glucose tolerance was classified according to the 2003 ADA recommendations where

normal glucose tolerance (NGT) was defined as FPG < 6.1 mmol/L and 2 h-PG < 7.8 mmol/L; impaired fasting glucose (IFG) was defined as 6.1 mmol/L ≤ FPG < 7.0 mmol/L and 2 h-PG < 7.8 mmol/L; isolated impaired glucose tolerance (I-IGT): FPG < 6.1 mmol/L and 7.8 ≤ 2 h-PG < 11.1 mmol/L; diabetes defined as FPG ≥ 7.0 mmol/L and/or 2 h-PG ≥ 11.1 mmol/L.

#### 2.2.3 I-IGT diagnosis and subject selection

In this study, subjects were classified based on the 2003 ADA criteria for glucose tolerance categories. I-IGT was defined as FPG < 6.1 mmol/L and 2h-PG between 7.8 and 11.1 mmol/L, while subjects with FPG ≥ 6.1 mmol/L were excluded to ensure that only individuals with normal fasting glucose levels were included. All subjects underwent OGTT to confirm glucose tolerance status. Those with FPG ≥ 7.0 mmol/L or 2h-PG ≥ 11.1 mmol/L were excluded to remove individuals with diabetes. The final dataset consisted of individuals with normal fasting glucose but elevated post-load glucose levels (I-IGT group) and those with both normal fasting and post-load glucose levels (NGT group).

#### 2.2.4 Medical history documentation

Patients’ medical histories and medication regimens were recorded.

#### 2.2.5 Study ethics

This study received approval from the Ethics Committee of the Chinese People’s Liberation Army General Hospital (S2015-038-01). All patient identity data remained confidential.

### 2.3 Data analysis

**Figure 1:**
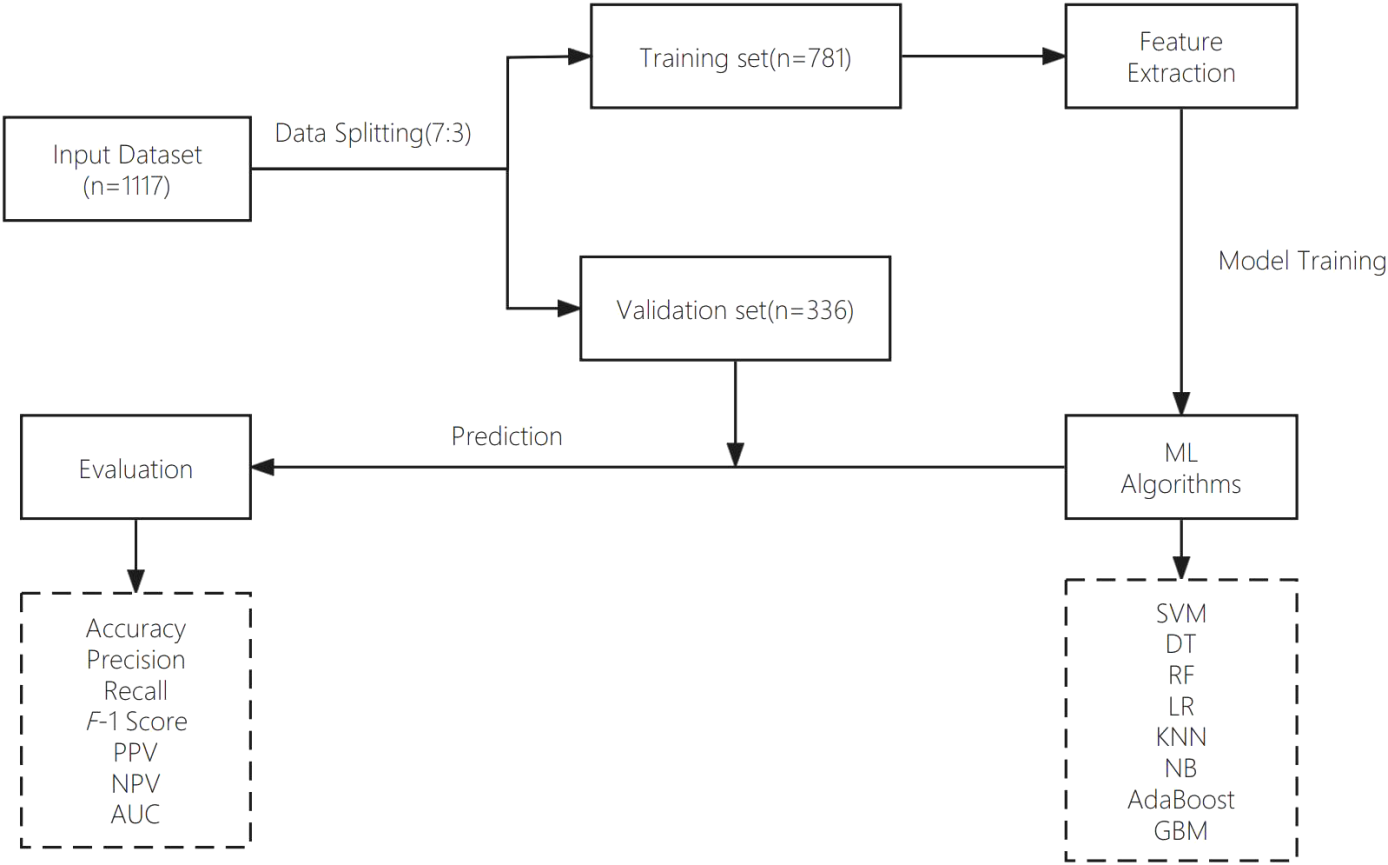
Proposed model workflow.

The dataset used in this study was retrospectively retrieved from the EMR system of the People’s Liberation Army (PLA) General Hospital. An overview of the different steps of data analysis is provided in this section. **Figure 1** presents the main workflow of this study.

#### 2.3.1 Data preprocessing

Clinical data from medical databases often contain incomplete and noisy information, making preprocessing a vital step in developing accurate predictive models. In this study, we employed listwise deletion to handle missing data, removing any observations with at least one missing value. This straightforward method requires minimal computation and assumptions, ensuring that only complete cases are used for analysis, thereby avoiding the potential errors or biases introduced by imputation techniques.

#### 2.3.2 Machine Learning models

##### 2.3.2.1 Support vector machine

SVM is a robust and versatile machine learning algorithm, particularly effective for classification tasks. It identifies the optimal hyperplane that separates different classes in the feature space, making it suitable for high-dimensional data. SVM’s capability to handle situations where the number of dimensions exceeds the number of samples makes it valuable in medical diagnosis, where it can classify patients based on complex medical data (15).

##### 2.3.2.2 Decision tree

DT recursively splits input data into subsets based on specific conditions, resulting in a tree-like structure. It is widely used in medical tasks due to its interpretability, as the model structure is easy to visualize and understand. However, DTs can be prone to overfitting, particularly with complex datasets (16).

##### 2.3.2.3 Random forest

RF is an ensemble learning method that constructs multiple decision trees and combines their outputs to enhance prediction accuracy and mitigate overfitting. RF has been used in predicting cardiovascular and Alzheimer’s diseases. Its main advantage is the ability to handle large datasets and high-dimensional feature spaces effectively, although it can be more complex and less interpretable than simpler models like decision trees (17).

##### 2.3.2.4 Logistic regression

LR is a statistical method for analyzing binary outcome variables, often used in modeling the probability of disease occurrence. LR is easy to interpret and understand, providing insights into variable relationships. However, its reliance on the assumption of linearity between predictors and the log odds of the outcome may not always hold in real-world situations (18).

##### 2.3.2.5 The K-nearest neighbors

KNN is a simple and effective machine learning method used for classification and regression tasks. In medical research, KNN has shown promising results in diagnostic medicine, such as heart disease prediction and diabetes diagnosis. It operates by identifying the ‘nearest neighbors’ of a data point in feature space to predict the class or value of that point (19–21).

##### 2.3.2.6 Naive Bayes

NB is a powerful classification method based on Bayes’ Theorem, assuming independence among predictors. It efficiently handles large datasets, making it valuable in medical research for disease diagnosis and predictive analytics in patient care (22).

##### 2.3.2.7 Adaptive boosting

AdaBoost enhances the performance of decision trees on binary classification problems by combining multiple weak classifiers into a strong one. It is useful in medical applications for improving diagnostic accuracy in tasks like image recognition or patient data analysis (23).

##### 2.3.2.8 Gradient boosting machine

GBM is an advanced technique for predictive modeling, sequentially adding predictors to an ensemble to correct its predecessors. GBM combines weak predictive models, typically decision trees, into a strong overall model. It is instrumental in analyzing complex datasets for disease prediction, patient outcome forecasting, and personalized medicine due to its versatility in handling diverse data types (24).

#### 2.3.3 Statistical analysis

In this study, stratified sampling was used to achieve an even distribution of features in both the training and validation sets. Subjects were divided into high and low FPG subgroups based on the median FPG value. Individuals from each subgroup were then allocated to the training and validation sets at a 7:3 ratio. A predefined random seed (random state=42) ensured consistency and reproducibility. This approach ensured balanced representation and prevented selection bias. To assess comparability, we compared the clinical characteristics of patients in the training and test sets. Before conducting statistical tests, we assessed the normality of continuous variables using the Shapiro-Wilk test. Variables that followed a normal distribution were analyzed using independent sample t-tests, while non-normally distributed variables were assessed using the Mann-Whitney U test. Categorical variables, such as hypertension status or blood pressure, were analyzed using the chi-square test or Fisher’s exact test, depending on sample size. The variables assessed included age, BMI, FPG, WHR, TC, TG, HDL-C, LDL-C, and hypertension status. The statistical results showed that all p-values were greater than 0.05, confirming that there were no significant differences between the training and test sets, ensuring a balanced dataset for model training and evaluation. The details of these findings are presented in **Table 1**. The statistical analysis for this study was performed using R software, version 4.2.2. Several R packages were employed to facilitate the development and evaluation of the diagnostic classification models, such as randomForest, rpart, stats, class, adabag, and pROC.

**Table 1:** Baseline demographic and clinical characteristics of the included patients.

Characteristics	Total (n=1117)	Training set (n=781)	Validation set (n=336)	P value
Age (years)	70 (50-87)	70 (50-85)	70.5 (50-87)	0.47
FPG (mmol/L)	4.81 ± 0.61	4.81 ± 0.62	4.83 ± 0.60	0.61
I-IGT, n (%)	382 (34.2%)	266 (34.1%)	116 (34.5%)	0.44
HBP, n (%)	654 (58.6%)	454 (58.1%)	200 (59.5%)	0.30
BMI (kg/m2)	25.04 ± 2.94	25.10 ± 2.91	24.88 ± 3.01	0.24
WHR	0.89 (0.86-0.92)	0.89 (0.86-0.92)	0.89 (0.86-0.92)	0.41
TC (mmol/L)	5.18 (4.63-5.77)	5.15 (4.62-5.72)	5.22 (4.64-5.89)	0.39
TG (mmol/L)	1.53 (1.17-2.00)	1.53 (1.17-1.98)	1.53 (1.14-2.05)	0.85
HDL-C (mmol/L)	1.21 (1.03-1.42)	1.21 (1.03-1.41)	1.21 (1.04-1.43)	0.80
LDL-C (mmol/L)	3.59 (3.11-4.15)	3.57 (3.12-4.12)	3.65 (3.11-4.24)	0.30

FPG, fasting plasma glucose; I-IGT, isolated impaired glucose tolerance; HBP, high blood pressure; BMI, body mass index; WHR, waist to hip ratio; TC, total cholesterol; TG, triglyceride; HDL-C, high-density lipoprotein cholesterol; LDL-C, low-density lipoprotein cholesterol.

#### 2.3.4 Model performance assessment

In medical classification models, several evaluation metrics are commonly used to assess performance and effectiveness. These metrics help determine the model’s ability to accurately predict and classify health conditions or patient groups. In this study, we adopted accuracy, recall, F1-Score, PPV, NPV, and AUC as evaluation metrics, described as follows:

Accuracy: Measures the proportion of correct predictions, including both true positives (TP) and true negatives (TN).

Accuracy = (TP + TN)/(TP + TN + FP + FN)

Recall (also known as Sensitivity or True Positive Rate, TPR): Measures the proportion of true positives identified among all actual positive instances.

Recall = TP/(TP + FN)

F1 Score: The harmonic mean of precision and recall, balancing both values

F1 Score = 2 * (Precision * Recall)/(Precision + Recall)

Positive Predictive Value (PPV): Measures the proportion of positive test results that are truly positive.

PPV =TP/TP + FP

Negative Predictive Value (NPV): Measures the proportion of negative test results that are truly negative.

NPV = TN/TN + FN

In addition to these metrics, we employed Receiver Operating Characteristic (ROC) curve analysis to evaluate model performance across different decision thresholds. The ROC curve plots the true positive rate (recall) against the false positive rate (1-specificity). The Area Under the ROC Curve (AUC-ROC) quantifies the model’s overall performance, with a score of 1 indicating perfect classification and 0.5 indicating random performance. Comparing AUC-ROC scores helps identify the most suitable model for the task.

By employing these evaluation metrics, we assessed the performance of our classification models and selected the most appropriate model for predicting and classifying health conditions. The use of multiple metrics provided a comprehensive evaluation of each model’s strengths and weaknesses, ultimately guiding clinical decision-making and enhancing patient care.

## 3 Results

**Figure 2:**
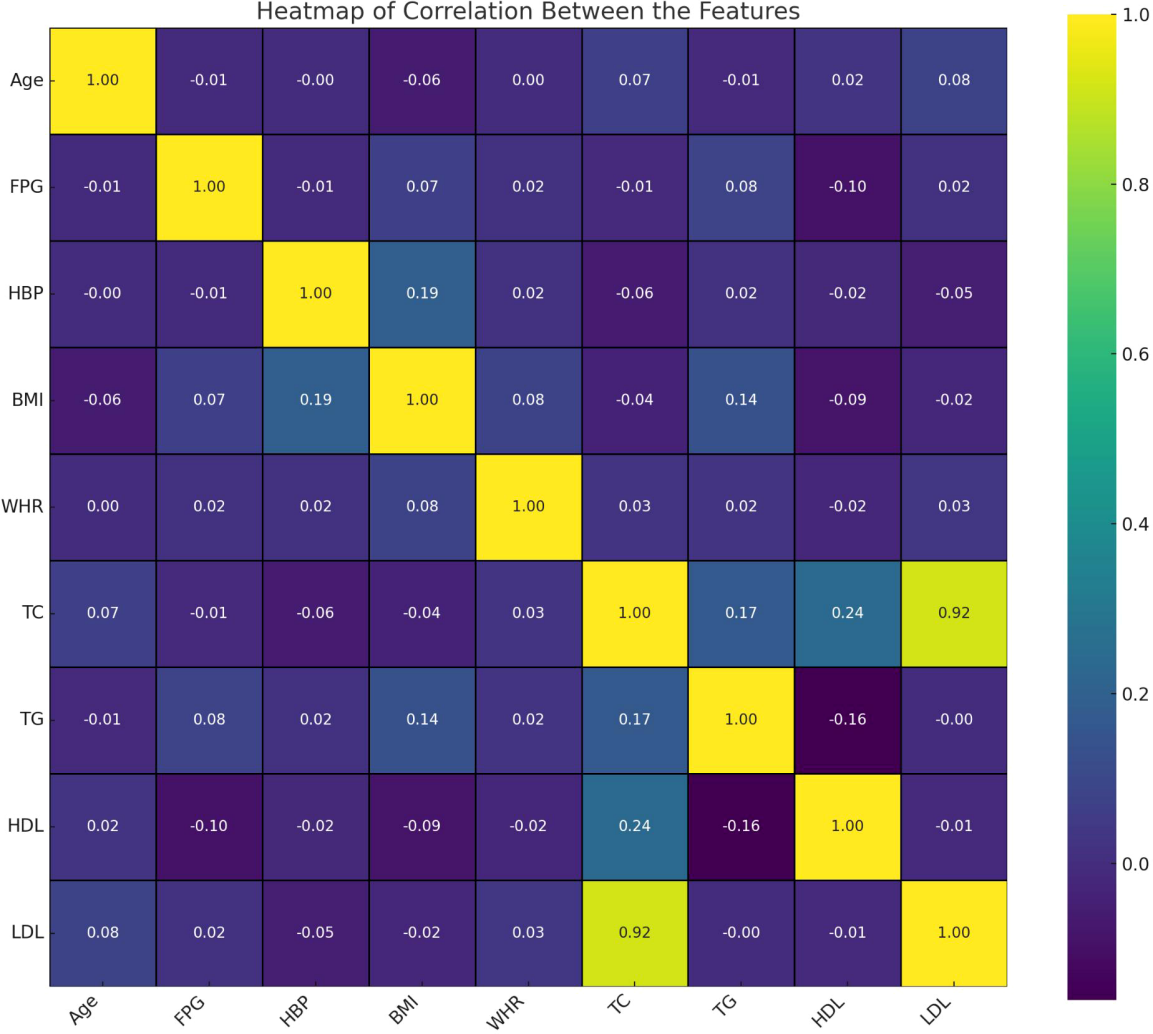
Correlation between the characteristics.

As described in **Table 1**, a total of 1,117 Chinese Han males with normal FPG levels was analyzed. The median age of the population was 70 (50-87) years, with fasting plasma glucose levels averaging 4.81 ± 0.61 mmol/L. Among the participants, 382 (34.2%) exhibited I-IGT, 654 (58.55%) had hypertension. The average body mass index (BMI) was recorded as 25.04 ± 2.94 kg/m^2^, and the mean waist-to-hip ratio (WHR) was 0.89 (range: 0.86 - 0.92). The overall lipid profile of the population indicated TC levels at 5.18 (4.63 - 5.77) mmol/L, TG at 1.53 (1.17 - 2.00) mmol/L, HDL-C at 1.21 (1.03 - 1.42) mmol/L, and LDL-C at 3.59 (3.11 - 4.15) mmol/L. The heatmap visualizes the correlation between features in this study (**Figure 2**). Each cell in the heatmap represents the correlation coefficient between two features. In this specific heatmap, we observe various degrees of correlation between features. A positive correlation was observed between BMI and WHR, indicating a link between overall and central obesity. Additionally, FPG was positively correlated with LDL-C, suggesting an interplay between glucose metabolism and lipid profiles. Notably, HDL-C showed a negative correlation with TG, which may reflect their inverse relationship in cardiovascular risk. Furthermore, a strong positive correlation was found between TC and LDL-C, emphasizing LDL-C’s significant role in cholesterol levels. Also, BMI showed a positive correlation with HBP, indicating a potential risk factor for high blood pressure.

Values for continuous variables are expressed as mean ± standard deviation or median [interquartile range] according to data normality; values for categorical data are given as number (percent). The P value represents comparison between training set and validation set.

### 3.1 Model performance comparisons

**Figure 3:**
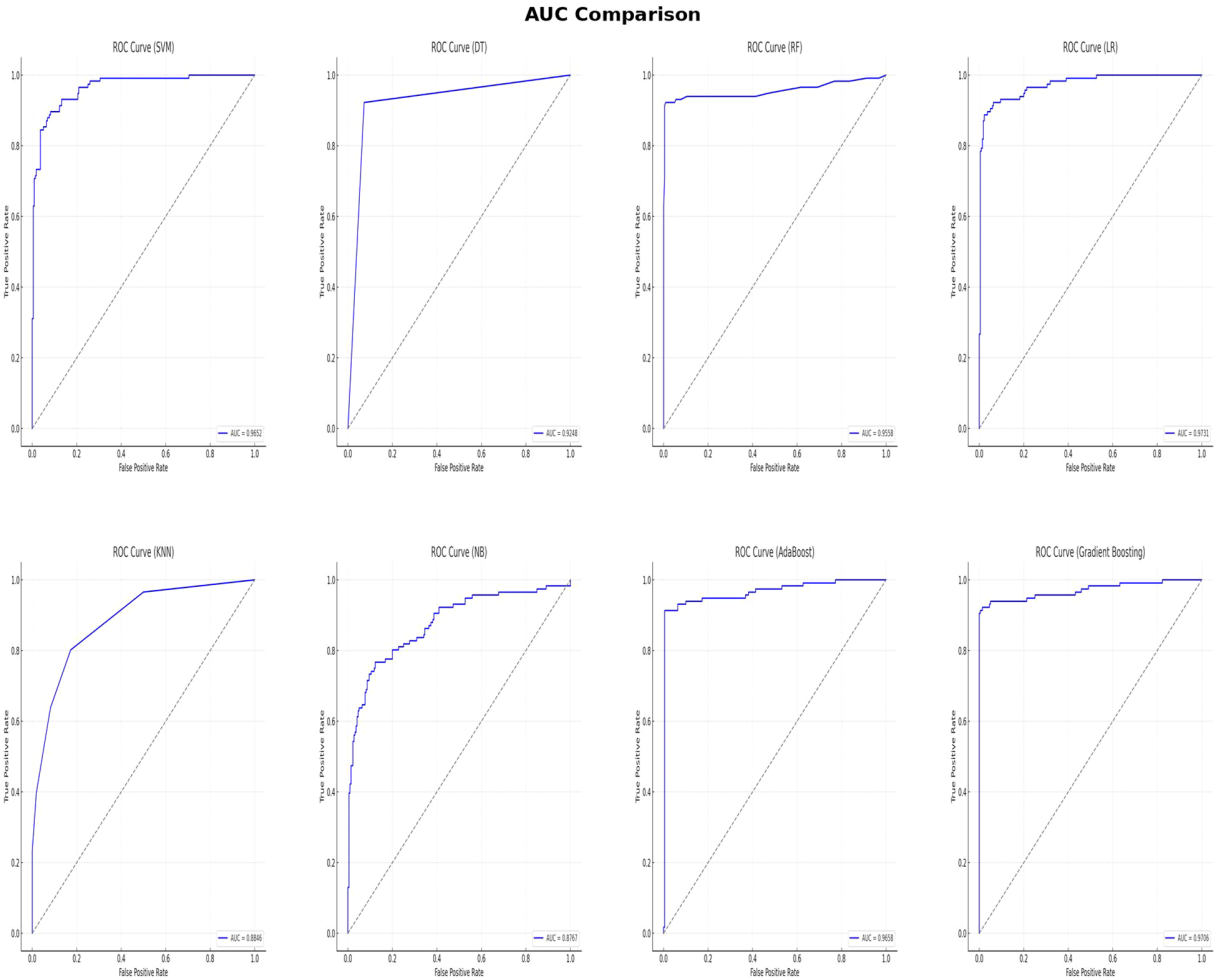
AUC comparison of eight machine learning algorithms.

In this study, multiple machine learning algorithms were employed to build predictive models. To rigorously evaluate the performance of each model, various metrics such as accuracy, recall, F1 score, PPV, NPV and AUC were employed. The results are summarized in **Table 2**. **Figure 3** clearly presents the ROC curves and AUC values of eight machine learning models. Among the eight models, the LR model (AUC=0.9731) performs the best, followed by the GBM model (AUC=0.9706), AdaBoost model (AUC=0.9658), SVM model (AUC=0.9652), RF model (AUC=0.9558), DT model (AUC=0.9248), KNN model (AUC=0.8846), and NB model (AUC=0.8767). Using the RF model as a reference, the LR, GBM, AdaBoost, and SVM models show superior performance, while the DT, KNN, and NB models exhibit inferior performance. Among the eight evaluated models, The LR model exhibited the highest AUC, while the RF model demonstrated the best accuracy (96.73%), recall (91.38%), F1 score of 95.7%, PPV of 99.07%, and NPV of 95.63%. These findings suggest that while the LR model may be more effective in distinguishing patients at risk, the RF models may offer a more balanced performance across various evaluation metrics.

**Table 2:** Model performance metrics.

Models	Accuracy	Recall	F1 Score	PPV	NPV	AUC
Support Vector Machine	90.8%	80.2%	85.7%	92.1%	90.2%	0.97
Decision Tree	92.6%	92.2%	89.5%	87.0%	95.8%	0.92
Random Forest	96.7%	91.4%	95.1%	99.1%	95.6%	0.96
Logistic Regression	93.5%	90.5%	90.5%	90.5%	95.0%	0.97
K-Nearest Neighbors	82.1%	63.8%	71.2%	80.4%	82.8%	0.88
Naive Bayes	67.9%	92.2%	66.5%	51.9%	93.1%	0.88
Adaptive Boosting	96.4%	91.4%	94.6%	98.2%	95.6%	0.97
Gradient Boosting	96.4%	91.4%	94.6%	98.2%	95.6%	0.97

PPV, positive predictive value; NPV, negative predictive value; AUC, area under curve.

**Figure 4:**
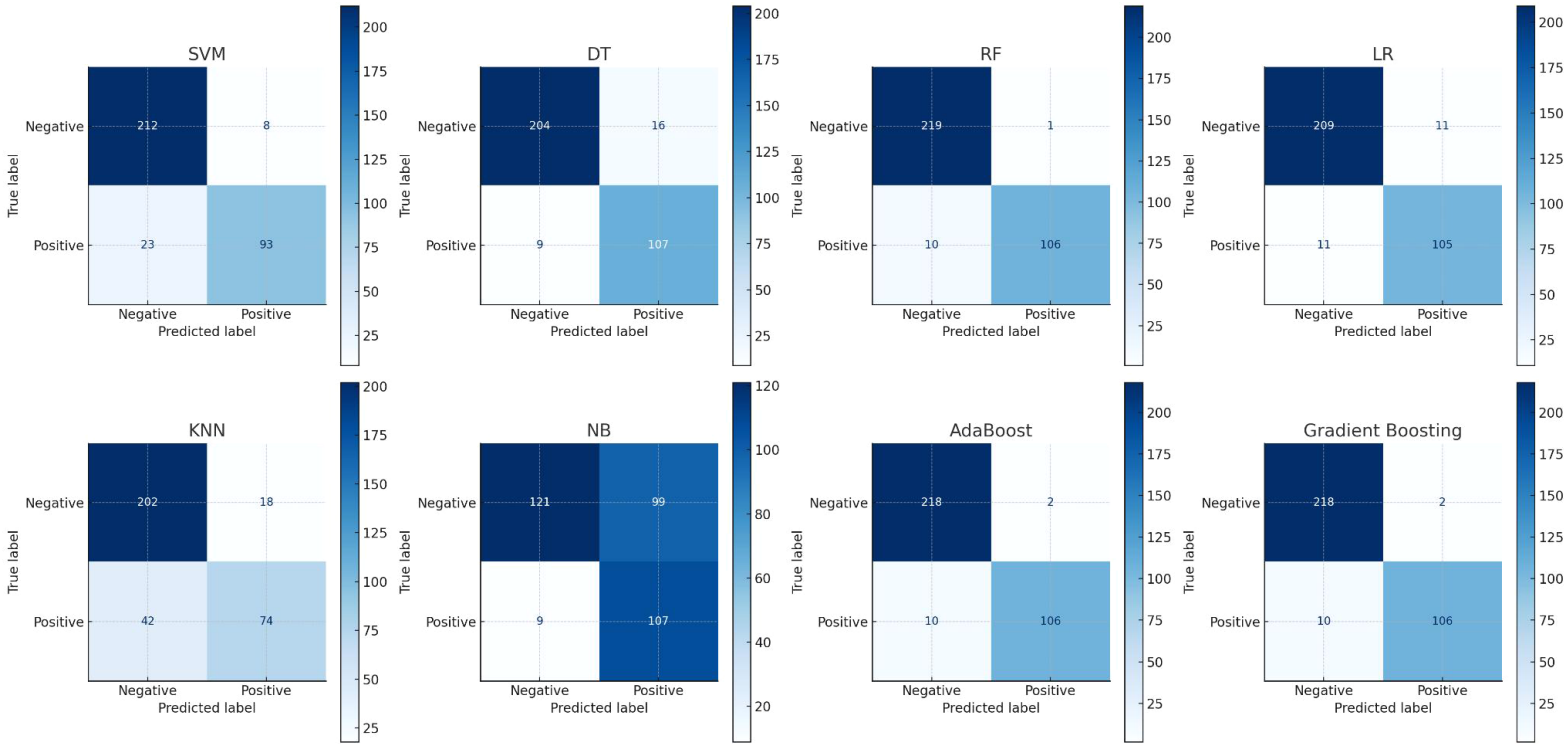
Show the eight confusion matrix to present the performances of applied eight machine learning algorithms where x-axis states the predicted level and y-axis states the true level.

The classification ability of various models is often demonstrated using confusion matrices, which are essential for evaluating model performance by displaying true positives, true negatives, false positives, and false negatives. Confusion matrices cross-tabulate actual outcomes with model predictions, providing insight into the accuracy and errors of the model. **Figure 4** presents confusion matrices for different machine learning models used in this study. The RF model shows a high number of true positives and true negatives with relatively few false positives and false negatives, indicating good performance. In contrast, the NB model has a higher number of false positives and false negatives, suggesting poorer performance compared to other models.

### 3.2 Explanation of risk factors

**Figure 5:**
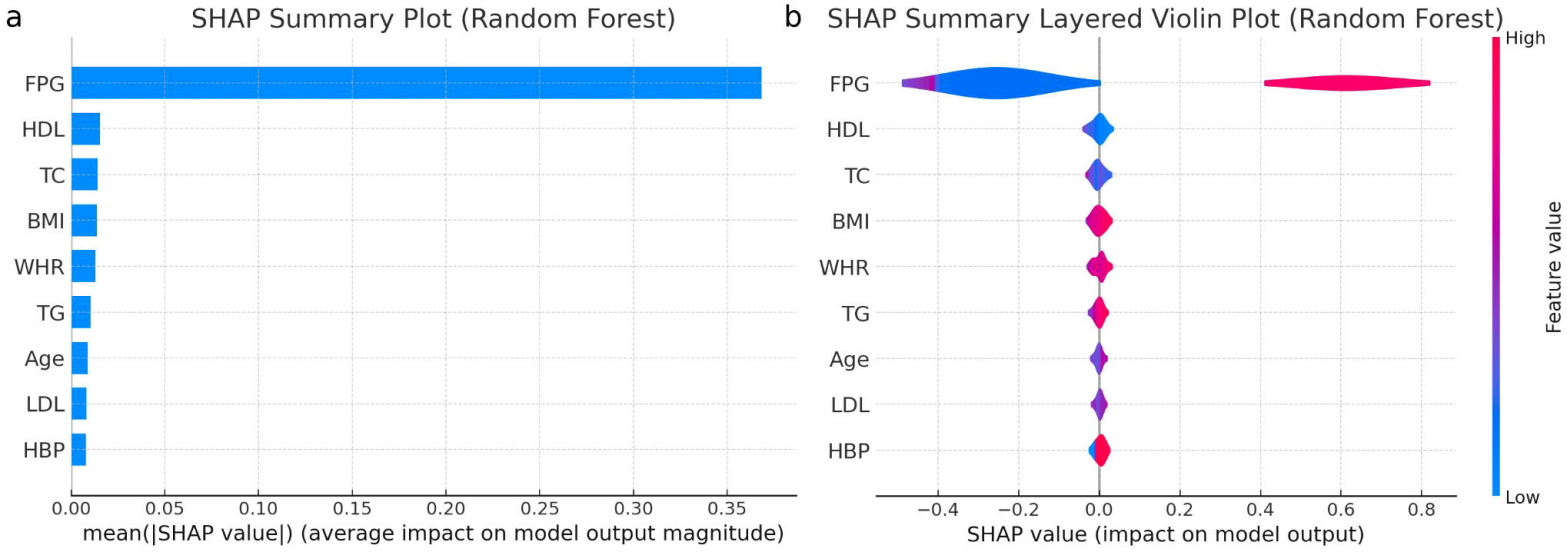
**(a)** Importance matrix plot of the Random Forest model, depicting the importance of each variable for predicting IGT in individuals with normal fasting plasma glucose levels. **(b)** SHAP summary plot of the 9 clinical characteristics of the Random Forest model. FPG, fasting plasma glucose; IGT, impaired glucose tolerance; T2DM, type 2 diabetes mellitus; HBP, high blood pressure; BMI, body mass index; WHR, waist to hip ratio; TC, total cholesterol; TG, triglyceride; HDL, high-density lipoprotein; LDL, low-density lipoprotein.

SHAP (Shapley Additive explanations) is used to explain the contribution of each variable in the model to the prediction outcomes. The RF model shows the highest TP and TN, **Figure 5** presents both the SHAP summary plot and the SHAP summary layer ed violin plot of the RF model. The SHAP summary plot ranks the predictive capabilities of variables such as FPG, age, BMI, WHR, TC, TG, HDL-C, LDL-C, and HBP for predicting I-IGT. The SHAP summary layered violin plot illustrates the distribution and impact of each feature on the RF model’s output.

Positive SHAP values push the prediction higher, while negative values push it lower. The width of the “violin” at each SHAP value level indicates the frequency of that impact value for the feature, with color indicating the actual feature. FPG emerges as the dominant predictor, with predominantly positive SHAP values, indicating that higher FPG levels significantly increase the likelihood of predicting glucose intolerance. Multiple analyses confirmed FPG as a potent predictor. These findings suggest that an FPG cut-off value of 5.1 mmol/L may effectively serve as a predictive indicator for I-IGT. The performance metrics for this cut-off included an accuracy of 89.35%, recall of 89.79%, F1 score of 85.22%, PPV of 81.09%, NPV of 94.38%, and AUC of 94.63%.

BMI demonstrates a broad distribution of SHAP values, implying varied effects on the prediction depending on the specific BMI value. WHR tends to influence the prediction towards normal glucose tolerance when lower, as indicated by its SHAP values concentrated in the negative range. TG, similar to FPG, mostly contribute positively, hinting that higher levels of TG are associated with an increased risk of glucose intolerance. HDL-C and TC show mixed impacts, with SHAP values distributed across both positive and negative, suggesting a nuanced influence on the model’s outcome. LDL-C predominantly falls on the negative side of SHAP values, suggesting that lower LDL-C levels might be linked to predicting normal glucose tolerance. Age has a relatively even distribution of SHAP values, indicating a less pronounced and more variable impact across different ages.

Finally, HBP shows a significant concentration of SHAP values in the negative range, suggesting that lower blood pressure readings might be predictive of normal glucose tolerance, though its overall impact is more subdued compared to factors like FPG and TG. The SHAP summary layered violin plot provides critical insights into the RF model’s behavior for each feature, aiding in model interpretation.

### 3.3 Applying the prediction model

**Figure 6:**
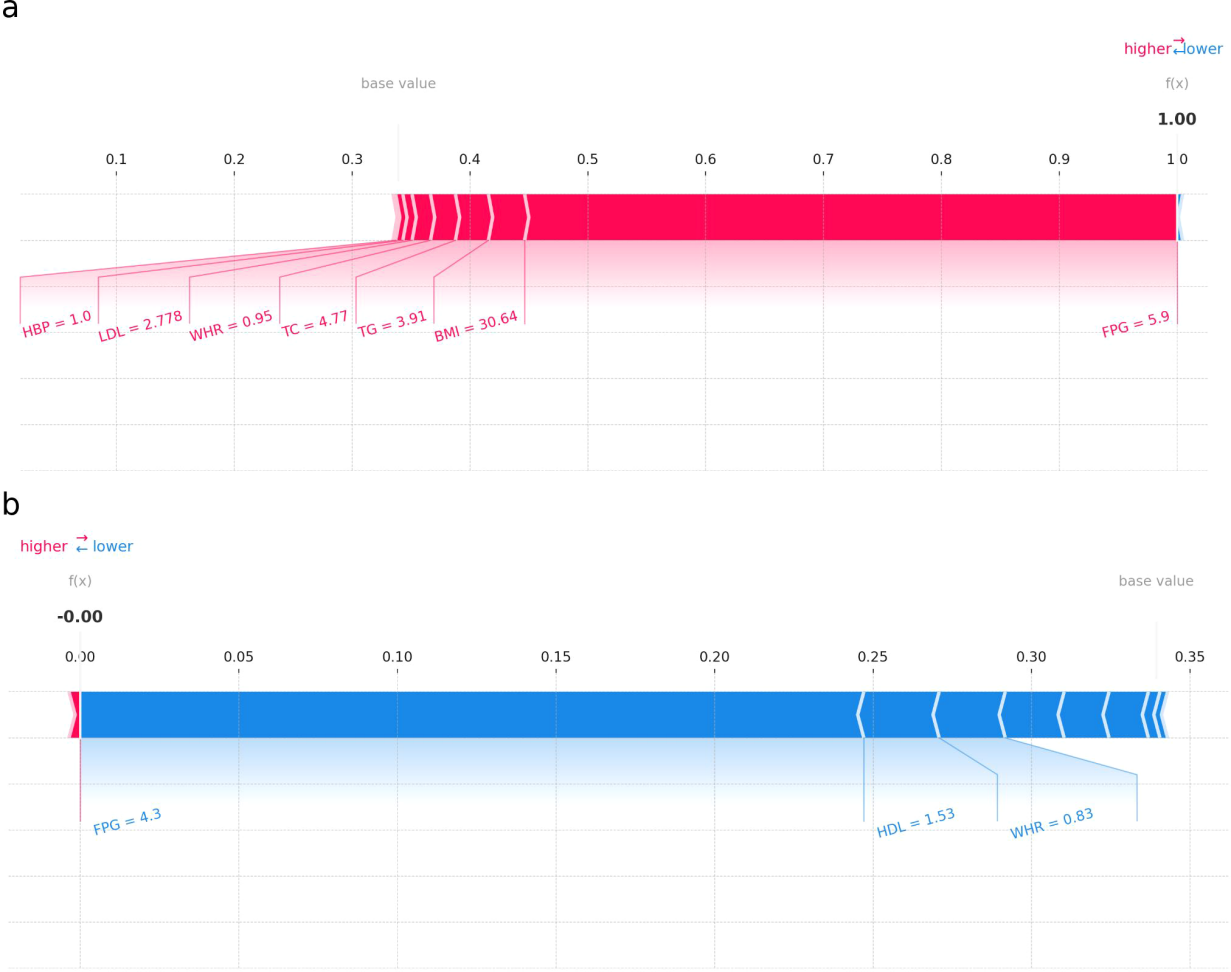
SHAP force plot for individuals in the dataset at high **(a)** or low **(b)** risk of IGT.

**Figure 6** presents two SHAP force plots for the RF model, labeled (a) and (b), providing insights into the model’s prediction behavior for two individuals. Plot (a) illustrates an individual with a higher probability of I-IGT. Each feature’s SHAP value is displayed as a bar, where the length and direction indicate the strength and direction of the feature’s impact. Features with positive SHAP values (extending to the right) increase the likelihood of impaired glucose tolerance. In this case, FPG, BMI, and TG have large positive SHAP values, indicating their strong contribution to the positive prediction. Plot (b) shows an individual with a prediction leaning towards a lower probability of I-IGT (shown in blue). Features such as FPG, HDL, and WHR have negative SHAP values, pushing the prediction lower. The most substantial negative impact is from FPG, as indicated by the length of its bar extending to the left. SHAP force plots are valuable for understanding the individualized predictions made by the RF model, demonstrating how each feature value contributes to the final prediction. This is crucial for interpreting the model’s decisions, ensuring transparency, and providing actionable insights in a clinical setting.

## 4 Discussion

In this study, we established eight predictive machine learning models to predictive male individuals with I-IGT, based on clinical variables from EMRs. The LR model exhibited the highest AUC, while the RF model demonstrated the best accuracy, recall, F1 score, PPV, and NPV. To our knowledge, this is the first published study to apply machine learning algorithms to predict I-IGT. Further analysis revealed a close relationship between FPG levels and I-IGT, with an FPG threshold of 5.13 mmol/L effectively distinguishing between NGT and I-IGT. This finding has significant implications for screening individuals with I-IGT.

Pre-diabetes is defined as an intermediate metabolic state between NGT and T2DM, which includes IGT and IFG, and poses a higher future risk for diabetes and/or cardiovascular diseases. Research indicates that prediabetes is more prevalent than diabetes (10). There is evidence to suggest that patients with prediabetes have a higher risk of cardiovascular diseases (25). The DECODE study group conducted an 11-year prospective study of nearly 30,000 non-diabetic participants from 22 European cohorts, revealing that when 2-hour glucose levels are between 7.8 mmol/L and 11.1 mmol/L, the risk of cardiovascular mortality begins to increase (26). Compared to IFG, diagnosing the IGT population requires a more time-consuming and complex OGTT, making early detection more challenging. The presence of prediabetic diabetes complications supports the necessity of timely screening and early intervention. However, there are currently no specific screening recommendations or guidelines targeting the IGT or I-IGT population. With the rising incidence of T2DM, numerous data mining techniques have been used to establish early disease diagnostic or predictive models, such as RF, LR, and Cox proportional hazards regression models. Models perform well in the specific population they were developed in but poorly in other datasets or populations, suggesting that developing specific predictive models for different populations or ethnicities might have better adaptability. Moreover, some predictive models incorporate too many feature parameters, making the models limited in practical application and difficult to widely disseminate (27). Our study employed eight models, with varying performances across models. KNN and Naive Bayes models showed poor performance, while RF, LR, and other models generally performed better, indicating that using machine learning to identify individuals with I-IGT is feasible and effective. To make the decision-making process of the models more understandable, tools like SHAP summary plots, SHAP layered violin plots, and SHAP force plots were used to provide a multi-faceted display of the models, which to some extent, increased physicians’ acceptance of the models.

Through the complex learning of multiple machine models, FPG was considered the most important indicator by all models, signifying a close connection between FPG and IGT has been identified through machine learning. Typically, FPG gives an indication of the baseline glucose level, while the OGTT provides insight into how well the body can regulate glucose after sugar intake, making them related but distinct measures of glucose metabolism. Our research revealed that using an FPG cut-off value of 5.1mmol/L effectively distinguishes individuals with I-IGT from those with NGT, suggesting that fasting glucose levels in the I-IGT population are higher compared to those with NGT. Rohit Babbar et al. conducted a study focusing on the use of machine learning to identify individuals with IGT without the reliance on an OGTT. The research demonstrated that machine learning methods have moderate accuracy in predicting glucose tolerance from a wide set of clinical and laboratory variables. Notably, fasting plasma glucose (FPG) was found to be the most important variable in all models, emphasizing its utility as a key predictive factor (28). This finding suggests that FPG, in combination with machine learning techniques, could facilitate a more efficient and less burdensome approach to early detection of IGT in at-risk populations. Kristina et al. found that a decline in pancreatic beta-cell function could be detected even among individuals with NGT as FPG levels increase, supporting the central role of beta-cell function in glucose regulation, especially when FPG levels are elevated (29). An analysis of data from over 12,500 participants revealed a graded relationship between FPG levels and glycated hemoglobin (A1C) levels, further emphasizing the importance of FPG as a predictive factor for glucose intolerance (30). Among individuals with NGT and IGT, there was a significant correlation between beta-cell secretory capacity and FPG levels, highlighting the importance of maintaining glucose homeostasis against rising FPG levels and pointing to the potential role of beta-cell function in the prevention and treatment of prediabetes and diabetes (31). Elevated FPG levels are often considered an early indicator of beta-cell dysfunction, reflecting decreased insulin sensitivity, which is closely associated with the development of T2DM (32). This finding underscores the complex physiological mechanisms behind glucose intolerance, involving multiple factors, indicating that changes in FPG levels may reflect alterations within this complex interaction network. The utilization of FPG as a solitary indicator for prediction offers the advantages of simplicity, ease of operation, rapidity, low technical requirements, and widespread acceptability. However, machine learning models consider multiple factors simultaneously, capturing complex relationships; provide higher predictive accuracy and personalized predictions; and demonstrate more stable performance, adapting better to data changes. Therefore, while FPG is sufficient for initial screening or in resource-limited settings, machine learning models are preferable for higher accuracy and comprehensive individualized assessments. In summary, the significant correlation between FPG levels and glucose intolerance underscores the role of various physiological mechanisms in the development of prediabetes and diabetes, offering potential for machine models to identify individuals with normal FPG but impaired glucose tolerance.

In addition to FPG, characteristics associated with metabolic syndrome such as TG, HDL, BMI, and WHR also played a role in model predictions. The SHAP summary layered violin plot demonstrated an association between higher TG levels and an increased risk of glucose intolerance. This finding aligns with previous research, which has indicated that elevated TG levels may contribute to the risk of glucose intolerance by inducing insulin resistance (33). The relationship between HDL cholesterol and the risk of T2DM is complex. Studies show that low levels of HDL cholesterol are associated with an increased risk of T2DM and prediabetes (34, 35). Another study conducted in China, the Beijing Longitudinal Study of Aging, found that individuals with higher HDL levels have a lower risk of T2DM (36). Our study found that the influence of HDL is complex, having both positive and negative impacts. We observed a wide distribution of SHAP values for BMI, suggesting that the impact of different BMI values on glucose intolerance varies among individuals. This finding aligns with previous research indicating that a high BMI is associated with an increased risk of T2DM, potentially influencing glucose metabolism through effects on insulin sensitivity and inflammatory states (37). However, our results also highlight that the risk of glucose intolerance at the same BMI level may differ among individuals, likely influenced by factors such as genetics, lifestyle, and physical activity levels (38). Furthermore, our analysis revealed that lower WHR were associated with predictions of normal glucose tolerance, with SHAP values primarily concentrated in the negative region. This suggests that a smaller WHR ratio may serve as a protective factor against glucose intolerance, consistent with literature identifying abdominal obesity as a risk factor for glucose intolerance and T2DM (39).

This study has several strengths. First, we utilized data from EMRs, which, compared to the stringent inclusion criteria of clinical trials, offers a more representative sample of the Chinese Han male population. Second, the study employed a variety of machine learning models, with both the LR and RF models showing excellent results. Comparatively, the RF model may provide a more balanced outcome across various model evaluation metrics. Third, our findings highlight the close relationship between FPG and glucose intolerance, while the relationships between body weight, WHR, lipid levels, age, and blood pressure with glucose tolerance are more complex, underscoring the complexity of assessing individual health status. Lastly, previous research on diabetes prediction models has mainly focused on identifying individuals with diabetes. In contrast, this study aims at identifying individuals with I-IGT, offering a novel approach that is particularly meaningful for early clinical screening of prediabetes.

Despite these strengths, our study has limitations. The male-only cohort and data sourced from a single hospital may affect the generalizability of our findings across different populations or ethnic backgrounds. Moreover, the focus on elderly males raises concerns about the model’s applicability to younger I-IGT populations. Additionally, while our sample size of 1,117 subjects is comparable to previous studies in diabetes risk prediction, a larger dataset could further improve model robustness and generalizability. Future studies should incorporate more diverse populations, multiple data sources, and a larger sample size to enhance the model’s generalizability. An external validation dataset is also required to assess the stability of our predictive models.

## 5 Conclusions

In conclusion, predicting isolated impaired glucose tolerance (I-IGT) in Chinese Han men over 50 years old based on baseline demographic and clinical characteristics using machine learning is a feasible technique. The constructed models demonstrate good predictive accuracy, with FPG identified as the most important predictor by all models. This approach may assist physicians in screening individuals who require further OGTT testing or in retrospectively identifying patients’ past I-IGT status, thereby facilitating early diagnosis and intervention for I-IGT.

## Data Availability

The raw data supporting the conclusions of this article will be made available by the authors, without undue reservation.
